# Repeat Self-Harm Following Hospital-Presenting Intentional Drug Overdose among Young People—A National Registry Study

**DOI:** 10.3390/ijerph17176159

**Published:** 2020-08-25

**Authors:** Caroline Daly, Eve Griffin, Elaine McMahon, Paul Corcoran, Roger T. Webb, Katrina Witt, Darren M. Ashcroft, Ella Arensman

**Affiliations:** 1 National Suicide Research Foundation, Cork 021, Ireland; evegriffin@ucc.ie (E.G.); e.mcmahon@ucc.ie (E.M.); pcorcoran@ucc.ie (P.C.); ella.arensman@ucc.ie (E.A.); 2 School of Public Health, University College Cork, Cork 021, Ireland; 3 NIHR Greater Manchester Patient Safety Translational Research Centre, University of Manchester, Manchester Academic Health Sciences Centre (MAHSC), Manchester M13, UK; roger.webb@manchester.ac.uk (R.T.W.); darren.ashcroft@manchester.ac.uk (D.M.A.); 4 Division of Psychology & Mental Health, Centre for Mental Health and Safety, School of Health Sciences, Faculty of Biology, Medicine and Health, University of Manchester, Manchester Academic Health Sciences Centre (MAHSC), Manchester M13, UK; 5 Orygen, Centre for Youth Mental Health, The University of Melbourne, Parkville, Victoria 3052, Australia; katrina.witt@orygen.org.au; 6 Division of Pharmacy & Optometry, Centre for Pharmacoepidemiology and Drug Safety, School of Health Sciences, Faculty of Biology, Medicine and Health, University of Manchester, Manchester Academic Health Sciences Centre (MAHSC), Manchester M13, UK; 7 Australian Institute for Suicide Research and Prevention, Griffith University, Queensland 4122, Australia

**Keywords:** self-harm, repeat self-harm, method switch, overdose, drugs, young people

## Abstract

Background: The incidence of hospital-presenting self-harm peaks among young people, who most often engage in intentional drug overdose (IDO). The risk of self-harm repetition is high among young people and switching methods between self-harm episodes is common. However, little is known about their patterns of repetition and switching following IDO. This study aimed to investigate repeat self-harm and method-switching following hospital-presenting IDO among young people. Methods: Data from the National Self-Harm Registry Ireland on hospital-presenting self-harm by individuals aged 10–24 years during 2009–2018 were examined. Cox proportional hazards regression models with associated hazard ratios (HRs), survival curves and Poisson regression models with risk ratios (RRs), were used to examine risk factors for repetition and method-switching. Results: During 2009–2018, 16,800 young people presented following IDO. Within 12 months, 2136 young people repeated self-harm. Factors associated with repetition included being male (HR = 1.13, 95% CI: 1.03–1.24), aged 10–17 years (HR = 1.29, 95% CI: 1.18–1.41), consuming ≥ 50 tablets (HR = 1.27, 95% CI: 1.07–1.49) and taking benzodiazepines (HR = 1.67, 95% CI: 1.40–1.98) or antidepressants (HR = 1.36, 95% CI: 1.18–1.56). The cumulative risk for switching method was 2.4% (95% CI: 2.2–2.7). Method-switching was most likely to occur for males (RR = 1.36; 95% CI: 1.09–1.69) and for those who took illegal drugs (RR = 1.63; 95% CI: 1.19–2.25). Conclusion: Young males are at increased risk of repeat self-harm and method-switching following IDO and the type and quantity of drugs taken are further indicators of risk. Interventions targeting IDO among young people are needed that ensure that mental health assessments are undertaken and which address access to drugs.

## 1. Introduction

The highest rates of self-harm (regardless of suicidal intent) are consistently seen among young people, and several countries have reported increases in youth self-harm in recent years [1,2,3,4,5,6,7,8,9]. These trends are of concern, considering the association between self-harm and increased risk of suicide in young people, with repeated self-harm further elevating this risk [10]. The risk of self-harm repetition is high among young people. Within 12 months of self-harm between 15–25% of people under 18 years represent to hospital with repeat self-harm [2,9,11,12]. One-third of all IDO presentations are made by people under 25 years [13,14] and IDOs are proportionally highest among adolescents aged under 18 years [15]. Compared to other methods of self-harm, IDO is associated with a lower risk of repetition. However, people who combine IDO with self-cutting have a 14–50% higher risk of repetition than those who use IDO alone [2,16,17]. Clinical factors associated with repeat IDO include recent inpatient psychiatric care, alcoholism and having a diagnosis of depression [14]. The consumption of multiple drug types or larger quantities of tablets and the involvement of psychotropic drugs—in particular benzodiazepines—are also associated with an increased risk of IDO repetition [18,19,20,21]. However, the evidence regarding the association between drug type and repetition risk is inconsistent [19,22].

Switching methods between self-harm episodes is common [10], with approximately one third of individuals (33.3%) switching self-harm methods between the index and repeat episode [23]. Upon reviewing existing research, we found that individuals most commonly switch from self-injury to self-poisoning and that there are no definitive patterns in terms of escalation to methods of potential lethality within the existing literature [10,23,24]. A recent multicenter study among 10–18-year-olds identified that switching to methods with increased lethality was common, specifically from self-poisoning to hanging or asphyxiation [10]. There are indications that individuals who use methods with high potential lethality (e.g., attempted drowning, gassing, firearms, hanging and jumping) are more likely to switch methods, than those who use methods with lower potential lethality (e.g., IDO and self-cutting) [15,25,26]. Method-switching varies by age, with 37% of people under 25 years switching their non-fatal self-harm method within an average of 2.8 years [15]. Identified factors associated with non-fatal self-harm method-switching include being male, having a suicide intent scale (SIS) score of greater than 15, being in receipt of inpatient or outpatient psychiatric treatment and having multiple previous self-harm presentations [24,27].

The knowledge base regarding repeat self-harm and method-switching following IDO is limited and no known published study has examined the drug-related factors associated with repeat self-harm and method-switching among young people. This study aimed to investigate repeat self-harm and method-switching following hospital-presenting IDO among young people. The objectives were: (i) to establish self-harm methods used by young people during the study period, (ii) to identify the demographic, clinical and self-harm characteristics of repeat presentations following IDO among young people and (iii) to examine methods used in non-fatal repeat self-harm presentations, including method-switching, following IDO among young people.

## 2. Materials and Methods

### 2.1. Setting and Sample

The data for this study were obtained from the National Self-Harm Registry Ireland (NSHRI). The NSHRI is a national surveillance system that monitors hospital-treated self-harm presentations to acute hospitals in the Republic of Ireland. We used data for the period 1 January 2007 to 31 December 2018—in line with the availability of data from all hospital emergency departments in Ireland. Self-harm is defined as ‘an act with non-fatal outcome in which an individual deliberately initiates a non-habitual behavior, that without intervention from others will cause self-harm or deliberately ingests a substance in excess of the prescribed or generally recognized therapeutic dosage and which is aimed at realizing changes that the person desires via the actual or expected physical consequences’ [28]. Information is collected by independently trained data registration officers (DROs) who identify self-harm presentations according to standardized operating procedures (SOPs). Intentional drug overdose presentations reported in this study refer to ‘non-fatal acts in which an individual intentionally ingests a drug with the intention to cause harm’. Presentations of IDO include those with 10th Revision International Classification of diseases codes for intentional injury or poisoning (ICD-10 codes of X60–64). Presentations of IDO exclude self-poisonings involving chemicals only (ICD-10 X66–69), alcohol only poisonings (ICD-10 X65), poisonings of undetermined intent (ICD-10 Y10–19), presentations of accidental overdose with medications prescribed to treat specific illnesses and overdoses with illegal drugs used for recreational purposes. Presentations of chemical poisonings are not classified as IDO but referred to as self-poisonings. Drug types taken in IDO can refer to both medications and illegal drugs. For the present study, presentations made by young people aged 10–24 years were included.

The study dataset was restricted to persons whose index self-harm episode occurred between 1 January 2009 and 31 December 2018. Individuals who presented with self-harm between 1 January 2007 and 31 December 2008 were excluded, as were their subsequent episodes. This method was used to identify as accurately as possible all individuals whose first episode, of self-harm occurred within the study period between 2009 and 2018. Inception cohorts for examination of self-harm repetition have been delineated in similar previously conducted studies [17,18,29].

### 2.2. Data Items

The data items examined for this study were: gender, age group (10–17 and 18–24 years), date of presentation, method(s) of self-harm, name and quantity of drugs taken, alcohol involvement, whether or not a mental health assessment was undertaken and recommended next care. Self-harm method was coded according to the ICD-10 codes for intentional injury or poisoning. Information on a maximum of five methods per presentation were recorded. Information pertaining to a maximum of 13 drugs taken in IDO was examined. The number of tablets taken in IDO reflects the total number of all tablets taken in an IDO presentation. Information on the drugs taken in IDO and the respective quantities are captured in the NSHRI via self-reported information from the patient, ambulance service records, hospital medical records and toxicology reports, if available.

Drugs taken in IDO were classified according to the anatomic therapeutic chemical (ATC) classification system, the detail of which can be found in the Guidelines for ATC Classification and DDD Assignment [30]. The ATC codes for the drug types reported are: analgesics ‘N02’, of which paracetamol ‘N02BE01’ is most common; opioids ‘N02A’, of which tramadol ‘N02AX02’ is most common; antiepileptics ‘N03’, of which pregabalin ‘N03AX16’ is most common; benzodiazepines ‘N03AE’, ‘N05BA’, ‘N05CD’ and ‘N05CF’, of which diazepam ‘N05BA01’ is most common; antidepressants ‘N06A’, of which escitalopram ‘N06AB10’ is most common; and anxiolytics ‘N05B’, of which diazepam ‘N05BA01’ and alprazolam ‘N05BA12’ are most common. Illegal drugs were ascertained using the Irish Misuse of Drugs Acts of 1977 and 1984 [31,32]. Multiple drug IDO refers to the involvement of two or more different drug types per presentation. Alcohol was not considered to be a drug type in this study.

### 2.3. Statistical Analyses and Reporting

Repeat self-harm was defined as a re-presentation to a hospital emergency department for any method of self-harm within the study period. Repetition of non-fatal self-harm by the same individual was identified via a unique identifier generated by the NSHRI. Next repetition episode was included in the analyses, which were conducted according to persons rather than presentations. Individuals’ follow-up time periods ranged from 1 day to 9 years and the cumulative risk of repeated self-harm was estimated at 12 months following the index episode.

Kaplan–Meier survival analyses were used to estimate the cumulative risk of repeat self-harm following and index IDO episode. Cox proportional hazard regression models were fitted to examine candidate risk factors for repeat self-harm, according to hazard ratios (HRs). Univariate analysis was first performed to identify variables associated with repeat self-harm. Variables included in the Cox models were those which have previously been shown to be associated with repeat self-harm risk [33], in addition to specific characteristics of the IDO presentations and its hospital management. Univariate analyses were also performed to identify variables associated with self-harm repetition with a different method, and variables with a *p*-value of < 0.2 according to a univariate analysis were subsequently included in the multivariate modeling [34]. Poisson regression models were fitted to examine differences in the profile and characteristics of individuals who repeated self-harm with the same method versus a different method. Risk ratios (RRs) were calculated, using the reference categories indicated in Table 2. Robust standard errors were used to calculate 95% CIs for RRs, using a modified Poisson regression approach [35]. Similar to the hazard regression models, variables with a *p*-value of <0.2 in a univariate analysis were included in the multivariate modeling. Analyses were conducted using STATA 12 IC, StataCorp LLC, College Station, TX 77845-4512, USA.

The reporting of this study conformed to the strengthening the reporting of observational studies in epidemiology (STROBE) statement: guidelines for reporting observational studies [36].

## 3. Results

### 3.1. Characteristics of Index and Repeat Self-Harm Presentations

During the study period there were 37,340 self-harm presentations made by young people, involving 26,085 individuals. Approximately two-thirds of presentations were made by females (63.1%; N = 10,603) or by individuals aged 18–24 years (62.2%; N = 10,443). Just over one-quarter (26.5%) of these presentations were for repeat episodes. The mean number of repeat presentations over the study period was higher for females than males (4.03, SD = 12.53; 2.35, SD = 4.96) and for individuals aged 18–24, compared those aged 10–17 years (4.39, SD = 12.24; 1.44, SD = 3.51). By the end of the study period the cumulative risk of presenting to hospital with repeat self-harm was 23.6% (95% CI 23.0–24.2). Within 12 months of follow-up, the cumulative risk was 13.9% (95% CI: 13.5–14.3). Intentional drug overdose and self-cutting was associated with the highest repetition risk (HR = 1.19, 95% CI: 1.01–1.40), followed by self-cutting alone (1.16, 95% CI: 1.02–1.31). IDO alone was associated with a significantly reduced risk of self-harm repetition compared to other methods, as shown in Supplementary Table S1 (0.85, 95% CI: 0.76–0.96).

### 3.2. Characteristics of Repeat Presentations Following an Index Episode of IDO

**Figure 1:**
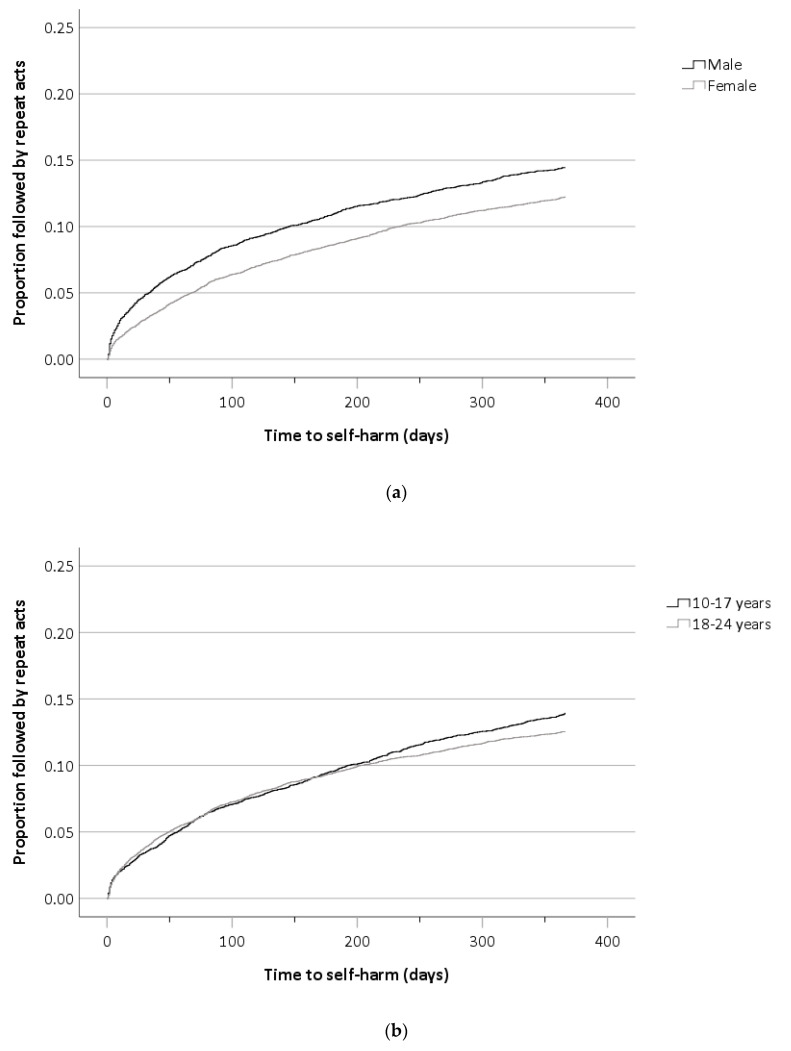

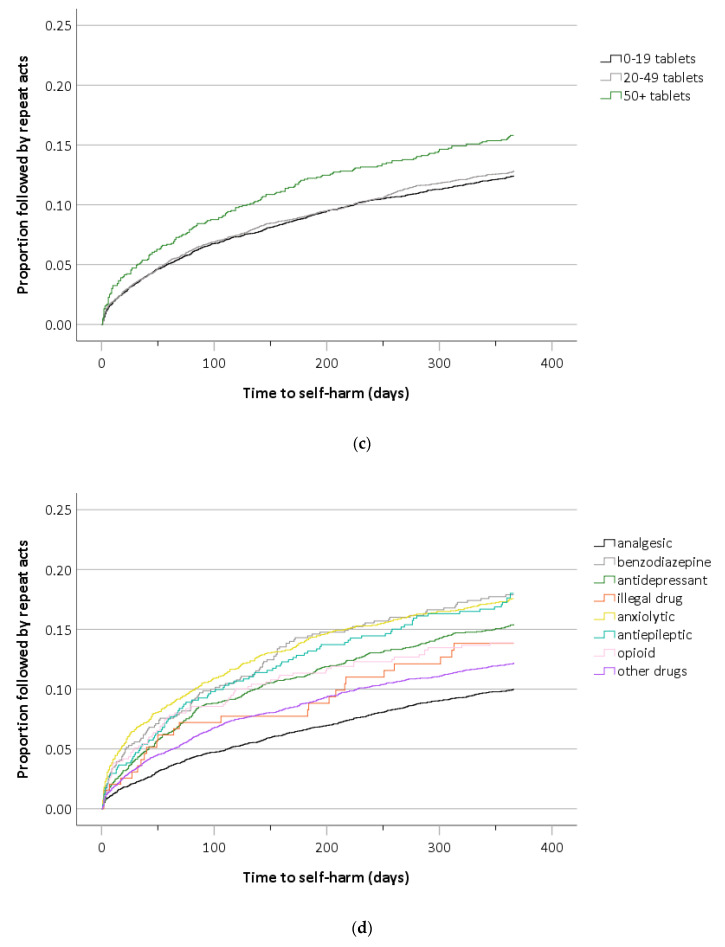
(**a**–**d**) Kaplan–Meier survival curves illustrating the cumulative risk of repeat self-harm within 12 months of the IDO episode. The risk of repeat self-harm presentation is illustrated by (**a**) gender, (**b**) age, (**c**) number of tablets taken and (**d**) type of drug taken.

Table 1 outlines the characteristics of individuals with an index IDO episode (N = 16,800) and those with a subsequent repeat self-harm episode with any method following IDO (N = 2136). By the end of the study period, the cumulative risk of presenting to hospital with repeat IDO was 18.2% (95% CI 17.5–18.9). Within 12 months of follow-up, the cumulative risk was 10.3% (95% CI 9.8–10.8). Repetition risk was higher for males compared to females (HR = 1.13, 95% CI 1.03–1.24) and time to repeat episodes were shorter for males, as illustrated in Figure 1a. Repetition was also higher for young people aged 10–17, compared to 18–24-year-olds (1.29, 95% CI 1.18–1.41). Repetition risk was higher among individuals who took 50 or more tablets in the index IDO, compared to those who took fewer than 20 tablets (1.27, 95% CI 1.07–1.49). Repetition occurred sooner for individuals who took 50 or more tablets in the index IDO (Figure 1c). Risk of repetition was significantly higher among individuals who took benzodiazepines (1.67, 95% CI 1.40–1.98) or antidepressant drugs (1.36, 95% CI 1.18–1.56) at their index IDO episode. In the univariate model, repetition risk was higher and occurred sooner among individuals who took anxiolytics (1.53, 95% CI 1.37–1.71) or antiepileptic (1.43, 95% CI 1.18–1.74) drugs in IDO and lower among those who took analgesic drugs (0.75, 95% CI 0.69–0.82). In the multivariate model, repeat self-harm was more common among individuals who combined IDO with self-cutting (1.37, 95% CI 1.20–1.58). Admission to a psychiatric ward or unit was the most likely recommended next care received following repeat self-harm (1.51, 95% CI 1.24–1.85). A mental health assessment was undertaken following almost three-quarters (74.4%) of IDO presentations. The likelihood of repeat self-harm following IDO was not impacted by the number of drug types an individual took in IDO, alcohol involvement or whether a mental health assessment was received by the young person. The multivariate model in Table 1 was also estimated for those with at least 12 months follow-up and the results were similar.

Table 2 describes the characteristics of individuals who engaged in repeat self-harm with IDO (N = 1758) and those who switched method (N = 378) within 12 months following an index IDO episode. By the end of the study period the cumulative risk of switching self-harm method following IDO was 4.7% (95% CI 4.3–5.1). The cumulative risk within 12 months was 2.4% (95% CI: 2.2–2.7). Method-switching was more likely to occur among males (RR = 1.36; 95% CI 1.11–1.66). Likelihood of method-switching varied depending on the type of drug taken in IDO, with individuals who took illegal drugs had the highest risk of switching (1.63; 95% CI: 1.25–2.14). In the multivariate model, the likelihood of switching method did not vary significantly by the number of tablets taken, alcohol involvement, recommended next care and whether or not a mental health assessment was undertaken at the index IDO episode.

### 3.3. Methods Used in Self-Harm Repetition

**Figure 2:**
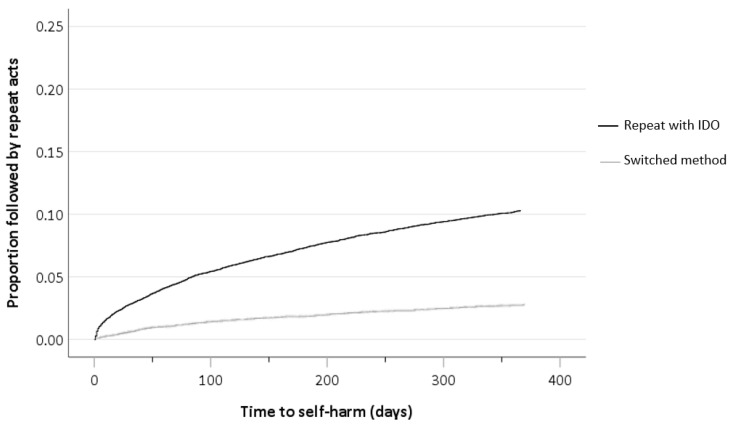
Cumulative risk of repetition within 12 months after index IDO episode according to repeat self-harm method.

Of the 2136 repeat self-harm presentations made following IDO, most young people repeated using the same method (1758, 82.3%). Figure 2 illustrates the cumulative risk of repetition with IDO or another method within 12 months, showing that repetition with IDO occurred more frequently and earlier than method-switching. Among those who switched method (N = 378), approximately two thirds of individuals used self-cutting in their next repetition episode (243; 64.3%). Switching to attempted hanging and drowning occurred for 16.4% (62) and 9.0% (34) of individuals, respectively. There were significant gender differences regarding methods switched to, with males switching to hanging more often than females (22.2% vs 10.3%, *p* = 0.002). Young people aged 18–24 years more often switched methods in their next repetition episode, compared to those aged 10–17 years (19.0% vs. 15.8%, *p* = 0.006). There were no significant differences in methods switched to according to the young person’s age.

## 4. Discussion

Using data on over 37,340 self-harm presentations by young people from a national registry, we have quantified the risk of repeat self-harm and method-switching among young people who presented with an index IDO episode. Within 12 months, the cumulative risk of repeat self-harm following IDO was 10% and factors associated with repetition included being male, aged 10–17 years, taking 50 or more tablets and taking benzodiazepines or antidepressant drugs in IDO. The risk of switching method at the next repeat episode was 2%, with most switching to self-cutting. Males and young people who took illegal drugs in IDO were most likely to engage in method-switching.

The recent increases in self-harm incidence among young people are particularly concerning considering that suicide risk is markedly elevated following self-harm [10]. It is important to note that the risk of suicide varies among young people, with older adolescents and young adults at greater risk of suicide than children and young adolescents [10,37,38]. The identified association between younger age (10–17 years) and increased risk of repeat self-harm is comparable to previous findings in Ireland [12]. Similar research also found greater repetition for young people aged 12–14 years, although the frequency of repetition was higher among those within the older age groups (22–24 years) [15]. This study identified that being male was associated with an increased risk of repetition and method-switching. There is little consensus in the literature in terms of gender-based differences in repetition risk, with our findings akin to some [15,39,40] and different from existing research [12,41,42]. Similarly, in relation to method-switching, males have been identified as at increased risk by this study and similar research [24]. However, an earlier study found a higher likelihood of method-switching among females [15]. The paucity of research with respect to differences in repetition risk according to gender and age signals the need for further research, with the purpose of informing targeted interventions to address gender-specific repetition risk.

To our knowledge, this is the first published study to identify drugs associated with repeat self-harm among young people. Between 66–82% of people of all ages who engage in IDO will do so using drugs that were prescribed to them [43,44]. However, this decreases to 55% in young people aged 10–34 years [43] and to 24% in adolescents (12–17 years) [45]. While the source of the drugs used in IDOs was unavailable for examination in this present study, it can be argued that efforts to monitor and limit access to prescribed drugs by young people are warranted. Measures which restrict access to means are underpinned by the integrated motivational-volitional (IMV) model of suicidal behavior, which asserts that access to means enables the transition from thinking about suicide to acting on those thoughts [46]. Clinically, the prescribing of antidepressants and benzodiazepines to children and adolescents should align with recommended guidelines [47,48] and the monitoring of adherence to medications is recommended. Furthermore, the Manchester self-harm rule cites taking a benzodiazepine in a self-harm episode as one of four key factors associated with repeat self-harm or suicide [20]. Despite increasing evidence that this and other risk assessment scales fail to predict future self-harm [49], the current findings illustrate a potential link between this drug class and repetition risk. When interoperating these findings, it is important to consider that medical diagnoses for which benzodiazepines or antidepressants are prescribed may have also conferred increased risk of repeat self-harm, which could not be accounted for using the NSHRI data.

Considering that young people are less likely to take their own prescribed medication in IDO [43,45] and are more likely to take over-the-counter (OTC) drugs than older people [50], the availability of all medications to young people needs to be addressed. In addition, the potential clinical severity of IDOs by young people is apparent in the findings of this study, with many taking multiple drug types in overdose, involving large quantities of tablets as well as illegal drugs. Public health measures to address the access by young people to large quantities of drugs that could potentially be taken in IDO are needed. Measures by which access to drugs can be successfully minimized include avoiding the prescribing of multiple drugs where possible [51], the safe storage and disposal of drugs [52,53], phased dispensing procedures [54] and legislation on the packaging and sales of particular drugs [55,56]. Furthermore, implementation of restrictive measures on illegal drugs can be effective in reducing drug-related psychiatric admissions and incidence of drug-related mental disorders [57]. Given the association between IDO with illegal drugs and elevated repeat self-harm risk, additional research is needed to identify effective preventive measures against use of illegal drugs by young people.

Several studies have examined method-switching following self-harm [10,23,24,29,58] and a previous review found that no discernible patterns in terms of escalation to methods of potential lethality can be identified [23]. The most common pattern of method-switching reported in many studies is from self-injury, including using methods with high potential lethality (e.g., drowning, hanging and jumping) [25,26], to self-poisoning [23]. While the risk of method-switching and repetition is lower for IDO than other methods [24], most people who die by suicide have used the methods of IDO or cutting in their index episodes [29]. This highlights the difficulties in assessing suicide risk according to methods used in self-harm. Within this study, 16% of young people who switched method after an index episode of IDO switched to attempted hanging in their next repetition episode and 9% to attempted drowning. These patterns—together with increasing trends in the use of methods with high potential lethality among young people [3]—are concerning as switching to a more lethal method in repeat self-harm is a significant predictor of suicide [26]. This and other research recommend that all young people who present to hospital following self-harm should receive a mental health assessment [23,59], in line with best practice guidelines [51,60]. Given that one quarter of young people who engaged in IDO in this study did not receive such an assessment, efforts to identify and address reasons for this are warranted. Considering the number of young people engaging in IDO, there is a need to ensure that evidence-based treatments and mental health service referral options are in place to address the needs of these young people within both hospital and community settings.

This is the first national study to examine repeat hospital-presenting self-harm and method-switching among young people following IDO, including over 37,000 self-harm presentations over a decade of observation. We used survival analyses to examine outcomes following IDO among young people. Analyses were confined to individuals with no self-harm presentations in the two years before the study period, to maximize the likelihood that first ever self-harm episodes were being examined. Several considerations must be accounted for when interpreting the study findings. It is possible that a small percentage of the identified inception cohort may have presented to hospital prior to 2007, therefore their episode of self-harm, recorded between 2009–2018, may not truly represent their first episode. Presentations to general practice only or self-harm episodes without presentation to healthcare services were excluded, thus limiting the generalizability of the findings to untreated self-harm within the community. Information on mental health history or psychiatric diagnoses are not recorded by the NSHRI, and so we were unable to adjust for these potentially important factors in our analyses. Furthermore, information recorded in the NSHRI is collected at the ED department and it is possible that some presentations could have resulted in death after presenting to the ED, which could not be accounted for. Information on drugs taken in IDO is collected via self-report by the presenting patient and this is supplemented by ambulance service records, hospital medical records and toxicology reports, where available. Finally, information on the source of tablets taken in IDO is not currently collected by the NSHRI and therefore it is unknown as to whether or not the individual was prescribed these drugs.

## 5. Conclusions

This national study examined the risk of repeat self-harm following IDO in young people, with a particular focus on patterns of method-switching. We found that young males were at increased risk of both repetition following IDO and method-switching—often to more potentially lethal methods of self-harm. We also identified that drugs such as benzodiazepines and illegal drugs were associated with risk of repetition and switching of methods among young people. The established risk of repeat self-harm among young people highlights the importance of ensuring that mental health assessments are provided to young people who present to hospital following self-harm. Furthermore, we surmise that an important intervention target is addressing the availability of both prescribed and over-the-counter drugs to young people. In conclusion, assuring the provision of mental health assessments and regulating drug access are key action areas for the prevention of suicidal behavior among young people.

## Figures and Tables

**Table 1:** Cox proportional hazards models indicating risk factors for self-harm repetition within 12 months following an index intentional drug overdose (IDO) episode.

Presentation Characteristics	Total Cohort: N = 16,800	Individuals Repeating with Any Method: N = 2136	Univariate Model		Multivariate Model ^f^	
	N	N (%)	HR (95% CI)	*p*	HR (95% CI)	*p*
**Gender**						
Male	6197	874 (14.1)	1.21 (1.11–1.31)	≤0.001	1.13 (1.03–1.24)	0.009
Female	10,603	1262 (11.9)	1.00	–	1.00	–
**Age**						
10–17 yrs	6357	862 (13.6)	1.12 (1.02–1.22)	0.012	1.29 (1.18–1.41)	≤0.001
18–24 yrs	10,443	1274 (12.2)	1.00	–	1.00	–
**Number of Drug Types ^a^**						
Single drug IDO	7901	1065 (12.0)	0.89 (0.81–0.96)	0.005	0.97 (0.88–1.06)	0.493
Multiple drug IDO	8897	1071 (13.6)	1.00	–	1.00	–
**Number of Tablets ^b^**						
0–19	6733	812 (12.1)	1.00	–	1.00	–
20–49	4984	623 (12.5)	1.03 (0.93–1.15)	0.543	1.05 (0.94–1.17)	0.391
≥50	1236	190 (15.4)	1.30 (1.11–1.52)	0.001	1.27 (1.07–1.49)	0.005
**Drug Type ^c^**						
Analgesic	7075	767 (10.8)	0.75 (0.69–0.82)	≤0.001	0.92 (0.79–1.07)	0.261
Benzodiazepine	3332	598 (17.9)	1.64 (1.50–1.81)	≤0.001	1.67 (1.40–1.98)	≤0.001
Antidepressant	2737	421 (15.4)	1.29 (1.16–1.44)	≤0.001	1.36 (1.18–1.56)	≤0.001
Anxiolytic	2238	391 (17.5)	1.53 (1.37–1.71)	≤0.001	0.97 (0.81–1.15)	0.709
Illegal drugs	1259	178 (14.1)	1.14 (0.98–1.33)	0.096	0.97 (0.82–1.15)	0.728
Antiepileptic	606	107 (17.7)	1.43 (1.18–1.74)	≤0.001	1.25 (1.02–1.54)	0.035
Opioid	552	75 (13.6)	1.08 (0.86–1.36)	0.517	–	–
Other drugs	5042	604 (12.0)	0.91 (0.83–1.00)	0.055	1.10 (0.93–1.30)	0.274
**Method/s**						
IDO alone	14,691	1789 (12.2)	1.00	–	1.00	–
IDO and self-cutting	1464	240 (16.4)	1.40 (1.22–1.60)	≤0.001	1.37 (1.20–1.58)	≤0.001
IDO and hanging	236	41 (17.4)	1.47 (1.08–2.00)	0.015	1.28 (0.93–1.76)	0.129
IDO and unspecified method	154	21 (13.6)	1.17 (0.76–1.80)	0.467	1.11 (0.71–1.71)	0.650
IDO and poisoning	99	16 (16.2)	1.36 (0.83–2.22)	0.224	1.25 (0.77–2.05)	0.365
IDO and drowning	61	8 (13.1)	1.15 (0.57–2.30)	0.697	1.09 (0.54–2.21)	0.811
**Alcohol Involved ^d^**	4534	577 (12.7)	0.99 (0.90–1.09)	0.897	–	–
**Recommended Next Care**						
Admitted to a ward	5556	728 (13.1)	1.10 (1.00–1.21)	0.046	1.09 (0.99–1.20)	0.079
Admitted to psychiatric ward or unit	567	110 (19.4)	1.67 (1.37–2.03)	≤0.001	1.51 (1.24–1.85)	≤0.001
Refused or left without being seen	1702	225 (13.2)	1.11 (0.96–1.28)	0.157	1.08 (0.93–1.25)	0.315
Not admitted	8975	1073 (12.0)	1.00	–	1.00	–
**Mental Health Assessment ^e^**						
No	2337	295 (12.6)	0.97 (0.85–1.11)	0.689	–	–
Yes	6501	842 (13.0)	1.00	–	–	–

^a^ Number of drug types was unknown for 2 (0.01%) of presentations, ^b^ Number of tablets was unknown for 3847 (22.9%) of presentations, ^c^ For each drug variable, the reference category is all presentations not involving that drug, ^d^ For the alcohol involved variable, the reference category is all presentations not involving alcohol, ^e^ Information on mental health assessment was collected from 2013 onward and was unknown for 715 of presentations (7.5%) made between 2013 and 2018, ^f^ Covariates included in the multivariate model include: gender; age; number of drug types; number of tablets; analgesic, benzodiazepine, antidepressant, anxiolytic, illegal drugs, antileptic; opioid and other drugs involvement; method/s and recommended next care.

**Table 2:** Characteristics of individuals who engaged in repeat IDO and those who switched method within 12 months of their index IDO episode.

Presentation Characteristics		Individuals Who Repeated N = 2136	Individuals Who Switched Method N = 378	Univariate Model		Multivariate Model ^d^	
		N	N (%)	RR (95% CI)	*p*	RR (95% CI)	*p*
**Gender**	Male	874	194 (22.2)	1.52 (1.27–1.83)	≤0.001	1.36 (1.11–1.66)	0.006
	Female	1262	184 (14.6)	1.00	–	–	–
**Age**	10–17 yrs	862	136 (15.8)	0.83 (0.69–1.01)	0.083	0.89 (0.73–1.09)	0.309
	18–24 yrs	1274	242 (19.0)	1.00	–		
**Number of Drug Types ^a^**	Single drug IDO	1065	207 (19.4)	1.22 (1.01–1.46)	0.057	1.25 (1.03–1.52)	0.045
	Multiple drug IDO	1071	171 (16.0)	1.00	–	–	–
**Number of Tablets ^b^**	0–19	812	124 (15.3)	1.00	–	–	–
	20–49	623	108 (17.3)	1.14 (0.89–1.44)	0.335	–	–
	50+	190	27 (14.2)	0.93 (0.63–1.37)	0.735	–	–
**Drug Type**	Analgesic	767	118 (15.4)	0.81 (0.66–0.99)	0.058	1.01 (0.79–1.31)	0.925
	Benzodiazepine	598	103 (17.2)	0.96 (0.78–1.18)	0.746	–	–
	Antidepressant	421	61 (14.5)	0.78 (0.61–1.01)	0.082	0.93 (0.70–1.24)	0.657
	Illegal drugs	178	54 (30.3)	1.83 (1.43–2.34)	≤0.001	1.63 (1.25–2.14)	0.003
	Anxiolytic	391	64 (16.4)	0.91 (0.71–1.16)	0.490	–	–
	Antiepileptic	107	19 (17.8)	1.00 (0.66–1.53)	0.988	–	–
	Opioid	75	11 (14.7)	0.82 (0.47–1.43)	0.526	–	–
	Other drugs	604	126 (20.9)	1.27 (1.05–1.54)	0.029	1.10 (0.86–1.41)	0.498
**Alcohol Involved**	Yes	577	113 (19.6)	1.15 (0.94–1.41)	0.208	–	–
**Recommended Next Care**	Admitted to a ward	728	125 (17.2)	1.02 (0.83–1.25)	0.879	–	–
	Admitted to psychiatric ward or unit	110	20 (18.2)	1.08 (0.71–1.64)	0.750	–	–
	Refused or left without being seen	225	52 (23.1)	1.37 (1.04–1.80)	0.045	1.23 (0.94–1.62)	0.191
	Not admitted	1073	181 (16.9)	1.00	–	–	–
**Mental Health Assessment ^c^**	No	295	49 (16.6)	0.92 (0.67–1.23)	0.612	–	–
	Yes	842	152 (18.1)	1.00	–	–	–

^a^ Number of drug types was unknown for 2 (0.09%) of presentations, ^b^ Number of tablets was unknown for 3847 (22.9%) of presentations, ^c^ Information on mental health assessment was collected from 2013 onward and was unknown for 715 of presentations (7.5%) made between 2013 and 2018, ^d^ Covariates included in the multivariate model include: gender; age; number of drug types; analgesic, antidepressant, illegal drugs and other drugs involvement; alcohol involvement and the recommended next care of refused or left without being seen.

## Supplementary Materials

The following are available online at https://www.mdpi.com/1660-4601/17/17/6159/s1, Table S1: Self-harm method at index episode and risk of repetition within 12 months.
