# Supplementary material for: Repeat Self-Harm Following Hospital-Presenting Intentional Drug Overdose among Young People—A National Registry Study
Source: Int J Environ Res Public Health. 2020 Aug 25;17(17):6159. doi: 10.3390/ijerph17176159 (PMC7504369; doi:10.3390/ijerph17176159)
Supplement: Supplementary file 1 [file ijerph-17-06159-s001.pdf]

Table S1. Self-harm method at index episode and risk of repetition within 12 months.

Method	All Individuals N = 26,085	Individuals Repeating N = 4383, n (%)	Univariate Model		Multivariate Model	
			HR (95% CI)	*p*	HR (95% CI)	*p*
**IDO only**	14,691	1789 (12.2)	0.84 (0.75–0.94)	0.002	0.85 (0.76–0.96)	0.007
**Self-cutting only**	5584	901 (16.1)	1.16 (1.03–1.31)	0.018	1.16 (1.02–1.31)	0.020
**IDO and self-cutting**	1464	240 (16.4)	1.18 (1.00–1.38)	0.052	1.19 (1.01–1.40)	0.037
**Attempted hanging only**	1360	163 (12.0)	0.83 (0.69–1.00)	0.052	0.82 (0.68–0.98)	0.034
**Attempted drowning only**	412	49 (11.9)	0.82 (0.61–1.10)	0.187	0.85 (0.63–1.15)	0.299
**Other method** [a]	2574	363 (14.1)	1.00	-	1.00	-

[a] Other method presentations include those with other known methods and those for which method is unknown.
